# Clinical trials in a COVID-19 pandemic: Shared infrastructure for continuous learning in a rapidly changing landscape

**DOI:** 10.1177/1740774520988298

**Published:** 2021-02-03

**Authors:** Haley Hedlin, Ariadna Garcia, Yingjie Weng, Ziyuan He, Vandana Sundaram, Bryan Bunning, Vidhya Balasubramanian, Kristen Cunanan, Kristopher Kapphahn, Santosh Gummidipundi, Natasha Purington, Mary Boulos, Manisha Desai

**Affiliations:** 1 Quantitative Sciences Unit, Division of Biomedical Informatics Research, Department of Medicine, Stanford University School of Medicine, Palo Alto, CA, USA; 2 Sean N. Parker Center for Allergy and Asthma Research, Department of Medicine, Stanford University School of Medicine, Stanford, CA, USA

**Keywords:** Platform protocol, Data Coordinating Center, efficient infrastructure, COVID-19, pandemic, Data & Safety Monitoring Board, master protocol, core protocol

## Abstract

**Background:**

Clinical trials, conducted efficiently and with the utmost integrity, are a key component in identifying effective vaccines, therapies, and other interventions urgently needed to solve the COVID-19 crisis. Yet launching and implementing trials with the rigor necessary to produce convincing results is a complicated and time-consuming process. Balancing rigor and efficiency involves relying on designs that employ flexible features to respond to a fast-changing landscape, measuring valid endpoints that result in translational actions and disseminating findings in a timely manner. We describe the challenges involved in creating infrastructure with potential utility for shared learning.

**Methods:**

We have established a shared infrastructure that borrows strength across multiple trials. The infrastructure includes an endpoint registry to aid in selecting appropriate endpoints, a registry to facilitate establishing a Data & Safety Monitoring Board, common data collection instruments, a COVID-19 dedicated design and analysis team, and a pragmatic platform protocol, among other elements.

**Results:**

The authors have relied on the shared infrastructure for six clinical trials for which they serve as the Data Coordinating Center and have a design and analysis team comprising 15 members who are dedicated to COVID-19. The authors established a pragmatic platform to simultaneously investigate multiple treatments for the outpatient with adaptive features to add or drop treatment arms.

**Conclusion:**

The shared infrastructure provides appealing opportunities to evaluate disease in a more robust manner with fewer resources and is especially valued during a pandemic where efficiency in time and resources is crucial. The most important element of the shared infrastructure is the pragmatic platform. While it may be the most challenging of the elements to establish, it may provide the greatest benefit to both patients and researchers.

## Background

Responding to the COVID-19 crisis requires a number of strategies including implementing social distancing policies to minimize community spread, increasing the ability of hospitals to safely manage COVID-19 cases, and understanding the epidemiology of disease progression and transmission. The response by researchers to the COVID-19 pandemic through the conduct of numerous clinical trials to identify effective treatment strategies has been unprecedented. As of June 2020, there have been over 2000 clinical trials related to COVID-19 registered through various international and national clinical trials registries including clinicaltrials.gov.^1^ This massive response and effort from researchers has pushed the existing clinical trial infrastructure to its limits and requires changes to the existing clinical trial system.^2^

Clinical trials are the gold standard for identifying effective treatments for disease and, therefore, play a crucial role in identifying strategies and interventions to resolve this pandemic. Standards for conducting trials in an ethical and scientifically rigorous manner have been developed both nationally and internationally.^3^ Specifically, good clinical practice (GCP) is defined as an international and scientific standard for the design, conduct, performance, monitoring, auditing, recording, analysis, and reporting of clinical trials. Following GCP is a detailed and involved process that consists of the following key activities listed in Table 1 and as described in the World Health Organization (WHO) handbook.^4^

**Table 1.** Activities involved in following good clinical practice.

Activity	Stage involved	Description of activity	Data Coordinating Center-specific Tasks
1	Launch	Development of the trial protocol	• Refine the question • Define the endpoint • Design the study including target population and frequency of measures • Establish the statistical analysis plan with well justified sample size
2	Launch	Development of standard operating procedures (SOPs)	• Establish SOPs for randomization, blinding, data management, and database lock
3	Launch	Development of support systems and tools	• Design electronic case report forms • Design, develop, and pilot test secure database capture
4	Launch	Generation and approval of trial-related documents	
5	Launch	Selection of trial sites and properly qualified, trained, and experienced investigators and study personnel	• Train coordinators across sites on the trial protocol, on using the database, and on data flow
6	Launch	Ethics committee review and approval of protocol	• Identify Data & Safety Monitoring Board (DSMB) • Hold kick-off meeting to get feedback on protocol
7	Launch	Review by regulatory authorities	
8	Conduct	Enrollment of subjects into study: recruitment, eligibility, and informed consent	
9	Conduct	The investigational product(s): quality, handling, and accounting	
10	Conduct	Trial data acquisition: conducting the trial Safety management and reporting	• Implement the data monitoring plan • Perform interim safety and/or futility/efficacy analyses • Engage the DSMB
11	Conduct	Monitoring the trial	
12	Conduct	Managing/monitoring trial data	• Implement data management plan
13	Conduct	Quality assurance of the trial performance and data	• Perform final data checks • Lock the database • Clean data
14	Dissemination	Reporting the trial	• Perform data analysis • Provide principal interpretation of findings

Each of these activities can be categorized into the launch, conduct, or dissemination of a trial. The last column describes the Data Coordinating Center–specific tasks corresponding to each activity, demonstrating a complex and typically lengthy process to yield valid findings. For example, preparing the infrastructure at a participating site involves establishing electronic case report forms, designing secure database capture, and training clinical coordinators to consent patients and enter data using the data capture system. Metzger-Filho et al.^5^ demonstrate that for Phase III breast cancer clinical trials, activating a site (time from regulatory approval to first recruited patient) can take on average 169 days. The implementation of the trial is similarly lengthy. For example, a report conducted by the US Food and Drug Administration (FDA) found that Phase III trials take anywhere from 1 to 4 years to complete.^6^ Zwierzyna et al.^7^ evaluated dissemination of findings for Phase II–IV studies and demonstrated that only 25% of studies disclose their findings within 1 year of completion. The median time to first public reporting, whether through direct submission to clinicaltrials. gov or publication in a journal, was 18.6 months. Dissemination lag was smaller for results submitted to clinicaltrials.gov (median: 15.3 months) compared to results published in a journal (median: 23.9 months). While breakthrough therapy drugs take a median development time of 4.8 years (from start of clinical testing to approval), the overall drug development process is typically completed within 8–10 years or more.^8,9^

Data Coordinating Centers play an essential role in the launch, conduct, and dissemination of trials (see last column of Table 1). Many of the activities involved in the launch focus on designing the study. The Data Coordinating Center is also involved in decision-making in response to unexpected issues that arise while the trial is ongoing. With regard to conduct, the Data Coordinating Center is largely involved in assessing data integrity and quality, for example, to determine whether there are differences by site in entering data or in measurement quality. During the trial, the Data Coordinating Center engages the Data & Safety Monitoring Board on whether the trial should continue as planned or terminate early which may involve performing interim safety and efficacy analyses. At the end of the trial, the Data Coordinating Center performs the primary analysis, provides principal interpretation, and facilitates prompt dissemination of findings in a reproducible manner. Typically, these are events that occur in sequential order.

However, the timeline in a pandemic is not sequential and is considerably expedited. There is a desperate need to streamline the conduct of trials, preferably in parallel, and to do so in a manner that uses a minimal number of participants and resources during the COVID-19 pandemic.^9,10^ In addition, the disease and treatment landscape is quickly evolving. Multiple trials of different interventions and study phases may be launched simultaneously at the same institution, motivating the need for a robust infrastructure to facilitate expeditious timelines for each trial. For example, development of case report forms may be ongoing while the database is being built; discussions with regulatory agencies on the endpoint, types of samples collected, and study design may occur *while* finalizing the protocol instead of *after*. Consequently, it may be necessary to identify Data & Safety Monitoring Board members even without an approved protocol so the Data & Safety Monitoring Board may convene prior to the first patient enrolled.

Scientific rigor is as necessary as ever during a pandemic to enable conclusive answers with high validity. However, the rapidity required to arrive at answers quickly can threaten scientific rigor and therefore the validity of the findings. The senior author directs a statistical group called the Quantitative Sciences Unit at Stanford University that serves as a Data Coordinating Center for several COVID-19 and non-COVID-19 clinical trials. As the Data Coordinating Center for COVID-19 related trials, our approach has been to provide an efficient shared infrastructure to aid Data Coordinating Centers in their activities related to the launch, conduct, and dissemination of findings for trials at Stanford and beyond in the midst of this and future pandemics.

## Methods

Our approach to increasing efficiency while maintaining rigor is to have a shared framework for all trials. The motivation is twofold: (1) to eliminate the need to rebuild infrastructure with each trial and (2) to leverage and build upon previous trials to arrive at a more robust study design for a given trial. Our shared infrastructure consists of the following elements described in greater detail below and listed in Supplementary Material (Table S1).

### COVID-19 endpoint registry

At the onset of the COVID-19 pandemic, validated endpoints were lacking for the newly emerging disease. This has been true for the COVID-19 pandemic. While the disease shares elements with other conditions including pneumonia and influenza, there are unique features to COVID-19 that need to be reflected in the endpoint. Indeed, some of the first trials launched included the definition of the primary endpoint as an adaptive feature (e.g. Beigel et al.^11^). As we began to design studies here at Stanford, we relied on the literature for endpoints that were previously utilized that would capture meaningful changes in disease progression. To more systematically capture all approved endpoints, we created a registry, hosted on the Society of Clinical Trials COVID-19 Research Resources Hub site (https://www.sctweb.org/covid.cfm) as well as at Stanford (https://stanford.io/covid19endpoints) that can be used by investigators designing studies at Stanford and beyond.

**Figure 1.**
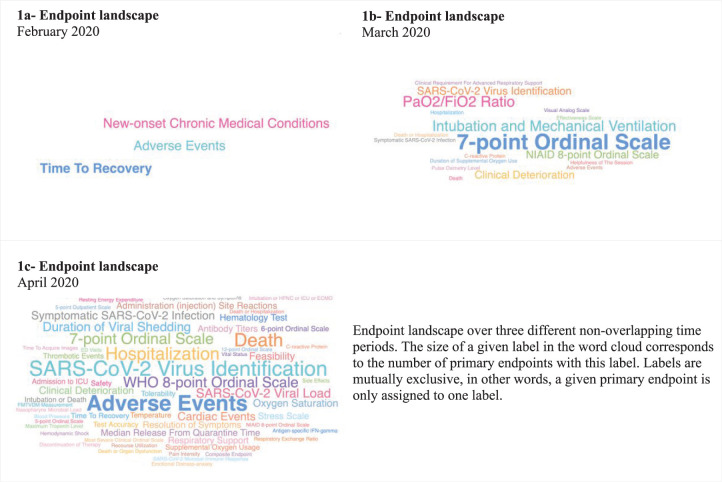
Endpoint landscape over time.

The main purpose of the registry is to provide guidance in designing studies by describing primary endpoints of studies conducted in the United States and listed on clinicaltrials.gov by various study design features. Endpoints are categorized into mutually exclusive families using methodology consistent with that of the COVID-19-modified version of Clinical Data Interchange Standards Consortium (https://www.cdisc.org/standards/therapeutic-areas/covid-19) and are given more refined descriptions so that more detailed guidance on endpoint selection can be provided. The registry provides information on study population, sample size, and how the use of endpoints changes over time. For example, the endpoints used at a particular time point can be graphically depicted using a word cloud, where the size of the word represents the popularity of the endpoint (Figure 1). Varying this over time would provide a visualization of how the endpoint landscape has changed over time. Figure 1(a)–(c) shows how the landscape altered from sparse in February 2020, at the onset when safety was a primary concern, to March 2020, when the ordinal endpoint was introduced for use in the hospitalized setting, to April 2020, when the diversity of endpoints demonstrated the launch of many more varied trials.

**Figure 2.**
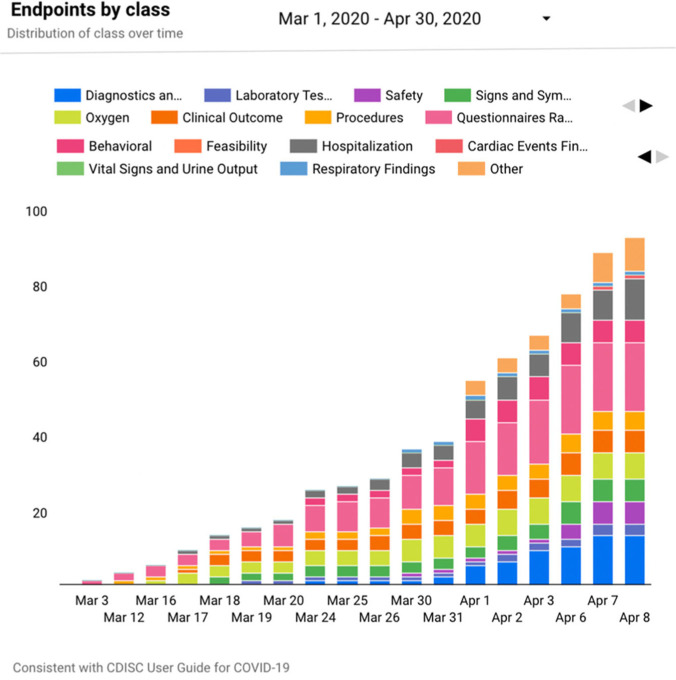
Endpoint class over time. The bars correspond to the total number of endpoint families on a given date. Each color corresponds to a specific endpoint class.

The distribution of endpoint families can also be visualized over time using other tools in the registry (Figure 2) that reflect how research priorities have shifted over time and furthermore which areas may be underrepresented.

**Figure 3.**
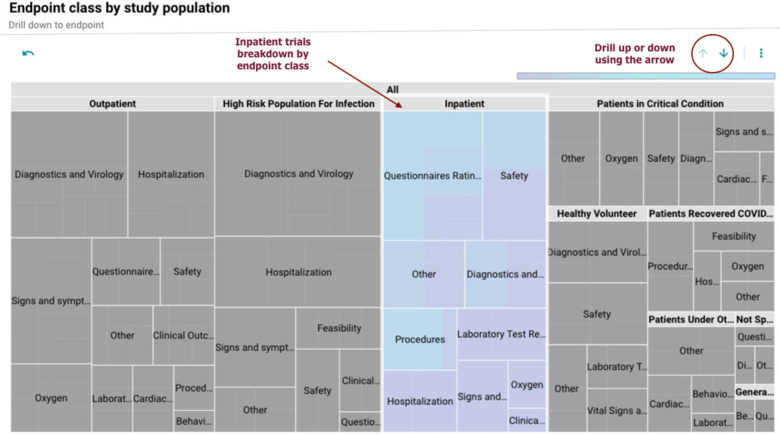
Endpoint class by study population. The size of the box corresponds to the number of primary endpoints for a specific population. Each box can be expanded to see additional details on the primary endpoints linked to it.

A treemap (Figure 3) allows the user to visualize how the endpoint classes are distributed across different target participant populations (inpatient, outpatient, etc.). The user can select a category of interest and drill up or down using the arrows at the top of the plot to collapse or view additional details, that is, which endpoints are present for the selected category. The box size also provides information on the number of primary endpoints linked to a specific population or research area.

One can further filter endpoints by specific characteristics related to the trial such as intervention type, study population, phase, and targeted enrollment. For example, a big challenge in designing trials for COVID-19 has been identifying endpoints relevant to the outpatient setting. One can use the registry to identify all endpoints used in this patient population. A search of the registry yields a list of all such endpoints, allowing one to link to the original clinicaltrials.gov registration to obtain more information.

### COVID-19 Data & Safety Monitoring Board registry

We developed an international registry to aid in identifying an appropriate Data & Safety Monitoring Board with diverse expertise and composition. The registry is hosted on the Society of Clinical Trials COVID-19 Research Resources Hub and at Stanford (https://med.stanford.edu/covid19/dsmb-registry.html). The registry lists experts who are interested in serving on (as a member or chair) and/or supporting (as an independent statistician or statistical group) one or more Data & Safety Monitoring Boards for trials studying interventions related to COVID-19. It is intended to be a tool to expedite Data & Safety Monitoring Board formation and to fulfill the unique Data & Safety Monitoring Board needs for COVID-19-related trials. The registry can serve non-COVID-19 trial needs as well and allows the user to search for members with specific expertise to ensure a representative composition which may include experts in virology, critical care, clinical trials monitoring, biostatistics, and ethics (Figure S1 of Supplementary Material).

### Shared documents, procedures, and data collection forms

As our team serves as the Data Coordinating Center for numerous clinical trials, we have an established archive of templates or examples of trial-related documents that facilitate the launch of new trials. This infrastructure was in place prior to the onset of COVID-19. However, tailoring the documents to address unique issues that arose in the launch of COVID-19 was necessary. Shared material now includes charters for Data & Safety Monitoring Boards; standard operating procedures for trial activities including blinding and unblinding members of the team, randomization, database management, and locking the database; statistical analysis plans; and data collection forms (Table S1). It is particularly important that individual COVID-19 trials have commonalities, such as data elements, timing of measurements, and type of samples collected, to allow meta-analyses or secondary analyses across pooled study cohorts. While each principal investigator may not be aware of other trials, our role as the Data Coordinating Center is to understand the entire landscape of related trials, as combining data are critically important. Therefore, it is necessary that much of the infrastructure including procedures and data capture be standardized, shared, and uniform. The analytic design including, for example, strata used in randomization and handling of missing data, should be as consistent as scientifically possible.

### Design and analysis team dedicated to COVID-19

An important component of the shared infrastructure is having a team that is dedicated to COVID-19. Members should gain familiarity with COVID-19-related trials conducted at their institution and beyond. Members meet at least weekly to update each other and share newly gained knowledge and experiences. Topics can range from understanding new FDA guidelines on constructing endpoints, newly released findings from clinical trials, or information on national trial activities to consolidate effort across academic medical centers. Such knowledge is key to ensuring that the trials conducted at their institution are appropriately informed by and build upon external work already conducted. Importantly, a dedicated COVID-19 design and analysis team enables shared knowledge across trials and increases efficiency by not having to rediscover systems already in place.

### A pragmatic platform protocol

A pragmatic platform protocol is the most important element of the shared infrastructure, allowing for common knowledge across various efforts to study agents in the population of interest.^12^ A platform protocol—also called a core or master protocol—enables simultaneous study of multiple agents or combinations of agents and can be implemented across multiple centers. At the onset of the current pandemic, Dean et al.^13^ emphasized the importance of such a protocol. The authors discussed its benefits, particularly in accumulating the evidence necessary to address the pandemic efficiently and expeditiously. Some widely publicized platform protocols of COVID-19 interventions include the Adaptive COVID-19 Treatment Trial or ACTT,^14^ SOLIDARITY,^15,16^ and RECOVERY trials^17^ and, as noted by Lane and Fauci,^18^ many of the most impactful findings about COVID-19 treatments have come from trials performed under a platform protocol. While not all trials can fit into this structure, many can, and the benefits outweigh the invested effort to establish such a protocol.^19^

There are a number of challenges involved in establishing a platform protocol.^20^ A significant challenge is gaining consensus on key design considerations among the various invested parties including the study team investigators, the pharmaceutical companies, and regulatory agencies. Design considerations include the drugs to consider, the endpoints, and determining how actions—such as dropping or adding an arm—should be made. Finally, solutions should be provided for anticipated logistical challenges including how patients are consented, what a placebo may look like when active arms are delivered in diverse ways, and how research coordinators need to be trained when a new drug is onboarded.

**Figure 4.**
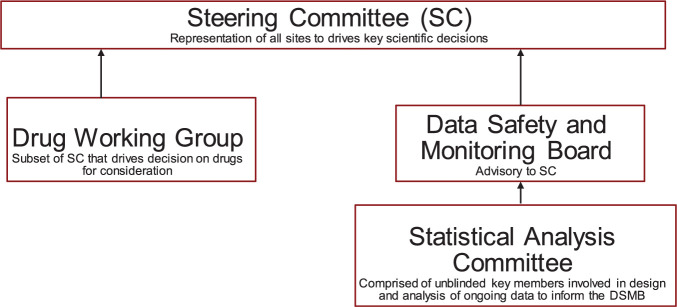
Possible governance structure for a platform protocol.

The governance and oversight for such a trial is therefore more complex and critical than in a standard trial. Governance should include four entities: a Steering Committee to drive key scientific decisions; a Drug Working Group, a subset of the Steering Committee, to help prioritize agents and combinations of drugs to consider; a single Data & Safety Monitoring Board to advise the Steering Committee; and a Statistical Analysis Committee to advise the Data & Safety Monitoring Board on ongoing safety and efficacy data (Figure 4).

## Results

As of this writing (November 2020) at Stanford, we have 40 total COVID-19 related trials pending or launched (14 of these are Stanford investigator-initiated, and 10 of the 40 trials are intended for the outpatient population). The Quantitative Sciences Unit serves currently as the Data Coordinating Center for six of the 14 Stanford-initiated trials.

Our infrastructure has been utilized for the six trials for which the Quantitative Sciences Unit has served as the Data Coordinating Center. Using our COVID-19 Data & Safety Monitoring Board registry which currently consists of over 50 members, we have established Data & Safety Monitoring Boards that share members across trials, ensuring shared knowledge across trials while allowing for differences in perspective. We have both contributed to the COVID-19 endpoint registry as our trials have received approval and benefited from the registry in identifying relevant endpoints for new trials.

A subset of the Quantitative Sciences Unit—15 members—comprises the COVID-19 Design and Analysis team. Members have a deep level knowledge of all COVID-19-related trials conducted at Stanford. In addition, two members have a dedicated role in debriefing the entire team on COVID-19-related trials and studies on a weekly basis. Such knowledge is key to ensuring that the trials we conduct at Stanford are appropriately informed by and build upon external work already conducted. Importantly, a dedicated COVID-19 design and analysis team enables shared knowledge across trials and increases efficiency by not having to rediscover systems already in place.

**Figure 5.**
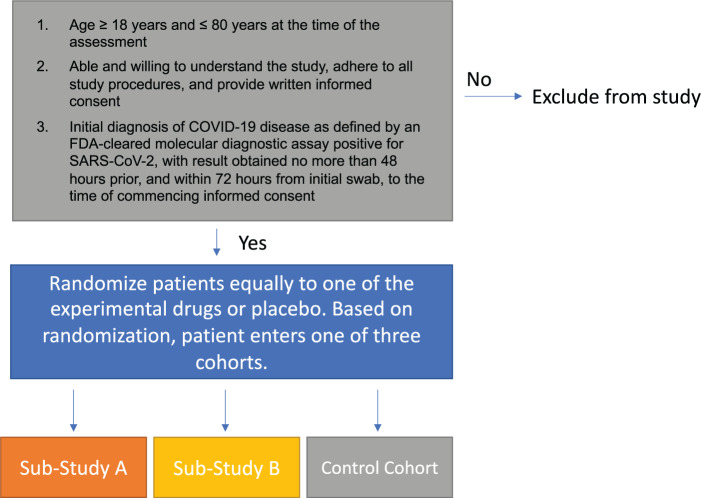
Schematic of adaptive platform trial for COVID-19 outpatients.

As of June 2020, the Stanford Quantitative Sciences Unit’s COVID-19 Design and Analysis team has established a flexible and pragmatic platform protocol with adaptive features for trials conducted in the outpatient setting. Originally, we had designed the trial to include hospitalized patients and treatments for the inpatient setting as well. However, because of national efforts to create platform trials in which Stanford could participate as a site (e.g. World Health Organization,^15^ REMAP-CAP,^21^ Foundation for the National Institute of Health),^22^ we revised our trial design to accommodate Stanford-initiated studies of drugs specifically for the outpatient setting (Figure 5). Trial characteristics include adaptive features with prespecified criteria that trigger actions including dropping or adding an arm and revising the randomization scheme accordingly. As per FDA guidelines on platform trials, there are no head-to-head comparisons across the active treatment arms.^23^ The pragmatic platform protocol is sufficiently flexible and includes two sub-studies that enable a virology based endpoint or a clinically driven endpoint depending on the primary interest regarding the agent, where data on both virology and symptomology will be measured in all trials. Trials may be stopped early for futility, harm, or efficacy. Among the many benefits of having a platform trial is the gain in efficiency. In addition to the common control group, gains in efficiency include one database capture system that has common standardized data elements and a single investigational new drug application that enables revision when new agents are added to the trial. The protocol has flexibility to allow variation in inclusion and exclusion criteria, so that patients who have a contraindication for a particular drug may still participate in the trial with a treatment assignment determined through randomization across the arms for which they are eligible.

## Conclusion

We have emphasized the importance of a shared infrastructure for launching and conducting clinical trials during the COVID-19 pandemic. This infrastructure enables substantial gains in efficiency and minimizes issues that may arise when expediency is valued but may threaten the rigor of the clinical trial.

A key component to the infrastructure is the pragmatic platform protocol. Importantly, a platform protocol resolves competition among trials for the same population, decreases the risk of trials not being completed, and in our proposed trial, reduces the number of participants needed by sharing controls. Thus, the pragmatic platform protocol addresses the ethical concerns that arise when multiple trials are launched simultaneously and resources are limited. In addition, the pragmatic platform protocol provides an appealing characteristic to the patient considering participation. In a single trial, the design often stipulates a 50% chance the patient will receive the placebo. A platform protocol with multiple active agents, even with a control arm, typically offers a less than 50% chance of receiving placebo as there are more active arms than there are inactive. In addition, other sites may be more interested in participating in a study where multiple agents are considered. This was particularly relevant at Stanford where the number of positive cases in the outpatient setting at the end of May was dwindling.

There are important statistical issues to consider when establishing infrastructure that relate to the design and analysis plan. Specifically, a robust study design with favorable statistical properties is key to yielding conclusive findings in trials and should feature prominently in any shared infrastructure. Modern designs that enable frequent looks at the data without paying the cost of Type I error have been established by numerous teams (e.g. ORCHID, REMAP-CAP).^24,25^ For example, the Outcomes Related to COVID-19 Treated with Hydroxychloroquine Among In-patients with Symptomatic Disease or ORCHID trial—a multi-center Phase III trial in the hospitalized setting with a targeted enrollment of 479 patients—conducted by the Prevention and Early Treatment of Acute Lung Injury (PETAL) Clinical Trials Network of the National Heart Lung and Blood Institute provided a blueprint for other teams to evaluate prespecified targets such as futility and efficacy nearly continuously. For increased efficiency, the trial relied on an ordinal endpoint that captures much more granular and clinically relevant information about a patient’s status at a given time point than a binary endpoint that simply measures death, for example. The investigators provide a detailed statistical analysis plan and code to implement their design, facilitating its adoption, and we encourage consideration of such designs.^26^

Importantly, ORCHID was able to provide definitive evidence that hydroxychloroquine did not provide benefit to patients hospitalized with COVID-19, a topic that had previously been a source of much controversy and confusion.^27,28^ Another example of a trial that relies on modern and favorable statistical properties is the large multi-center international platform trial: Randomized Embedded Multifactorial Adaptive Platform for Community-acquired Pneumonia or REMAP-CAP,^25^ which—in addition to Bayesian adaptive characteristics—holds a particularly interesting randomization feature called response-adaptive randomization that weights assignment toward those interventions that appear most favorable using posterior probabilities that are updated throughout the trial. While the platform for REMAP-CAP was already in place prior to the pandemic to study patients admitted to an intensive care unit with severe community-acquired pneumonia, it was sufficiently flexible to accommodate questions specific to the COVID-19 pandemic through a few design changes including those related to eligibility criteria and outcome. The outcome used for studying COVID-19 patients is a composite endpoint of in-hospital mortality and the duration of intensive care unit-based respiratory or cardiovascular support. As the goal of our Phase II study was to fully characterize either viral load or symptomology over time in a mild-to-moderate outpatient population, our most immediate priority was the ability to join the cohorts generated by each trial and to gain efficiency and shared knowledge across the individual trials and thus we selected a design that precludes frequent looks unlike the ORCHID or REMAP-CAP trials. An important advantage of our design, however, is that one large cohort of COVID-19 outpatients will be generated with commonly measured variables, facilitating numerous secondary analyses that pool information across sub-studies.

Prioritization committees have been established within academic medical centers to consolidate efforts, gain efficiency, and investigate the most promising avenues for treatment. Although the role of the Data Coordinating Center is not to prioritize which trials go forward, the shared infrastructure addresses these prioritization issues by allowing for more drugs to be evaluated efficiently while conserving the efforts of researchers. If multiple agents are evaluated under one randomized trial, such committees do not have to prioritize which drugs should be investigated. Therefore, a platform protocol should receive stronger consideration over a single trial as it involves a consolidated effort with much thought into key design considerations.

The infrastructure we established has provided a framework for distributing shared resources among trials conducted simultaneously by our statistical group. However, we have encountered challenges. As noted by others, the rapid pace of research during the COVID-19 pandemic has stretched researchers to extremes.^29–31^ Our shared infrastructure has alleviated some burden, but ongoing efforts to simultaneously conduct numerous trials still demand much of our statistical team, including those who facilitate non-COVID-19 research. Due to fiscal consequences of COVID-19 including hiring freezes imposed on the institution, employing additional team members is not a viable solution. Another challenge is the difficulty of aligning principal investigators, pharmaceutical companies, and other stakeholders on timing the launch of the platform protocol. Many investigators and drug sponsors have wanted to launch their individual trial instead of waiting to gain consensus among a larger group of investigators. While barriers to establishing the pragmatic platform protocol involve gaining consensus on key design considerations—which drugs to study, governance, timing, and endpoints—another obstacle is the lack of willingness to participate in the process and of recognition of the long-term value of consolidated effort. As of this writing, we are happy to report that our leadership and colleagues see the value of the pragmatic platform and are collaborating with us and regulatory agencies to launch a platform protocol in the immediate future.

The shared infrastructure provides appealing opportunities to evaluate disease in a more robust manner with fewer resources. Even in the absence of a platform protocol, a shared infrastructure allows resources to be used efficiently and data to be pooled, enabling secondary and subset analyses. While the resources we presented were tailored to Stanford’s environment, collaborative principles may be borrowed across institutions. Indeed, whenever possible the infrastructure developed at one institution should be shared across institutions as well. This can be in the form of shared protocols, analysis plans, registries, electronic case report forms (e.g. as made accessible by the Society of Clinical Trials COVID-19 Research Resources Hub, https://www.sctweb.org/covid.cfm) in addition to multi-center platform protocols. Ultimately, navigating the pandemic will require widespread sharing of information which can be done most efficiently through shared infrastructure. COVID-19 is neither the first global pandemic nor will it be the last. Thus, investment in the infrastructure we describe will also establish a platform for facilitating trials during future pandemics. Thus, the infrastructure itself should be designed to enable ongoing continuous learning that can be leveraged to facilitate trials within pandemics more generally.

## Supplemental Material

sj-pdf-1-ctj-10.1177_1740774520988298 – Supplemental material for Clinical trials in a COVID-19 pandemic: Shared infrastructure for continuous learning in a rapidly changing landscape Supplemental material, sj-pdf-1-ctj-10.1177_1740774520988298 for Clinical trials in a COVID-19 pandemic: Shared infrastructure for continuous learning in a rapidly changing landscape by Haley Hedlin, Ariadna Garcia, Yingjie Weng, Ziyuan He, Vandana Sundaram, Bryan Bunning, Vidhya Balasubramanian, Kristen Cunanan, Kristopher Kapphahn, Santosh Gummidipundi, Natasha Purington, Mary Boulos and Manisha Desai in Clinical Trials

sj-pdf-2-ctj-10.1177_1740774520988298 – Supplemental material for Clinical trials in a COVID-19 pandemic: Shared infrastructure for continuous learning in a rapidly changing landscape Supplemental material, sj-pdf-2-ctj-10.1177_1740774520988298 for Clinical trials in a COVID-19 pandemic: Shared infrastructure for continuous learning in a rapidly changing landscape by Haley Hedlin, Ariadna Garcia, Yingjie Weng, Ziyuan He, Vandana Sundaram, Bryan Bunning, Vidhya Balasubramanian, Kristen Cunanan, Kristopher Kapphahn, Santosh Gummidipundi, Natasha Purington, Mary Boulos and Manisha Desai in Clinical Trials
