# Supplementary material for: Clinical trials in a COVID-19 pandemic: Shared infrastructure for continuous learning in a rapidly changing landscape
Source: Clin Trials. 2021 Feb 3;18(3):324–34. doi: 10.1177/1740774520988298 (PMC8172421; doi:10.1177/1740774520988298)
Supplement: sj-pdf-1-ctj-10.1177_1740774520988298 – Supplemental material for Clinical trials in a COVID-19 pandemic: Shared infrastructure for continuous learning in a rapidly changing landscape [file sj-pdf-1-ctj-10.1177_1740774520988298.pdf]

Table S1. Elements of Shared Infrastructure

**Element**	**Description**
COVID Endpoint Registry	Registry of all primary endpoints considered for trials in the US pending or granted IRB-approval
COVID DSMB Registry	International registry of 1) DSMB members available for COVID studies and of 2) individuals willing to support DSMBs as the independent statistician or statistical group
Charters	Charters for DSMBs and Steering Committees with text addressing issues unique to COVID
Standard Operating Procedures	Documents describing procedures related to DSMB coordination, blinding, randomization, data management, and database lock
Statistical Analysis Plans (SAPs)	A standardized analytic plan for analysis with common data elements that allow for merging of data requiring variables to be uniform and similarly ascertained
Database Capture and Electronic Case Report Forms	Shared data elements and standardized capture of variables
Design and Analysis Team Dedicated to COVID	A team devoted to COVID-related studies familiar with issues unique to COVID, endpoints used and sources of variation in data generated
Platform Protocol	A protocol that enables the simultaneous evaluation of multiple agents or combinations of agents for the same disease and study population
