# Supplementary material for: Clinical trials in a COVID-19 pandemic: Shared infrastructure for continuous learning in a rapidly changing landscape
Source: Clin Trials. 2021 Feb 3;18(3):324–34. doi: 10.1177/1740774520988298 (PMC8172421; doi:10.1177/1740774520988298)
Supplement: sj-pdf-2-ctj-10.1177_1740774520988298 – Supplemental material for Clinical trials in a COVID-19 pandemic: Shared infrastructure for continuous learning in a rapidly changing landscape [file sj-pdf-2-ctj-10.1177_1740774520988298.pdf]

Figure S1. Number of registered experts by area of expertise

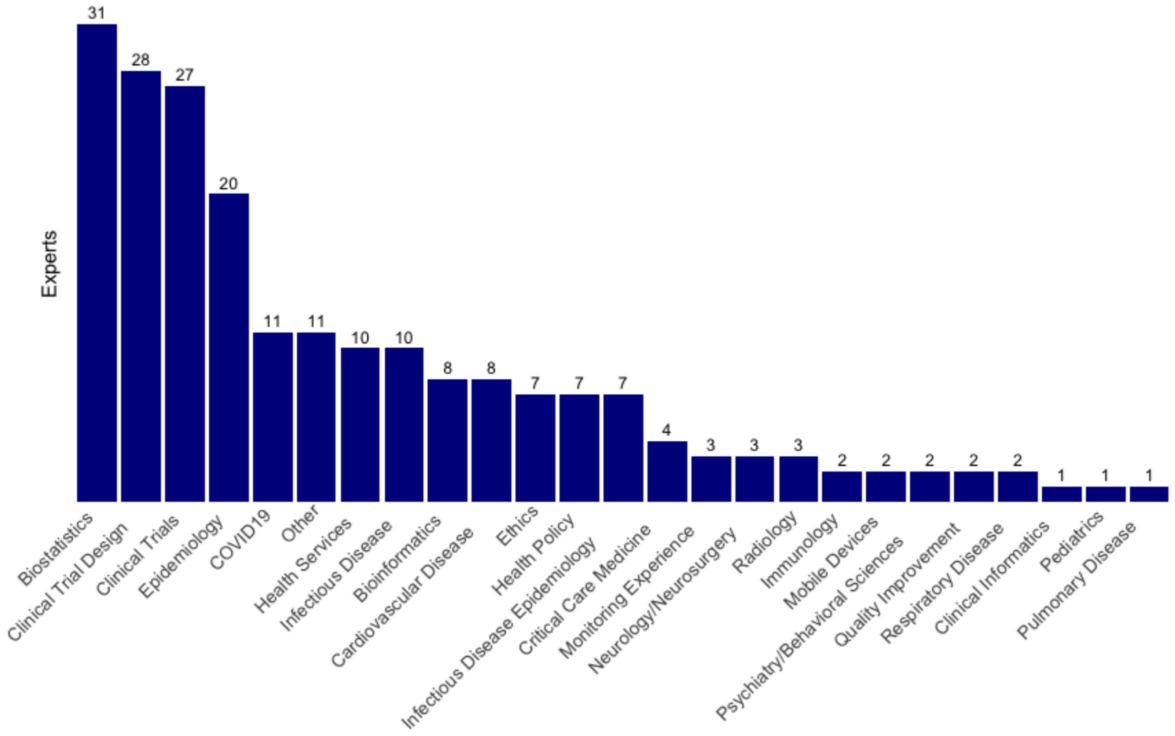

Note: Experts can indicate multiple areas of expertise
